# A microcomputer automated recording spectropolarimeter

**DOI:** 10.1155/S1463924679000584

**Published:** 1979

**Authors:** Victor C. Zadnik, James L. Scott, Robert Megargle, Julius Kerkay, Karl H. Pearson

**Affiliations:** Department of Chemistry Cleveland State University Cleveland Ohio 44115 USA


### A microcomputer automated recording spectropolarimeter

**Victor C. Zadnik, James L. Scott, Robert Megargle, Julius Kerkay, and Karl H. Pearson***

*Department of Chemistry, Cleveland State University, Cleveland, Ohio 44115*

MANY analytical laboratories have available minicomputers or microcomputer systems which routinely find use for data analysis. Recent trends have been toward applying computers not only for computational capabilities, but also for on-line control and data acquisition from actual analytical instrumentation. Numerous analytical instruments are being designed to incorporate a computer system as an integral part. However it is possible, in many cases, to economically adapt existing instrumentation to a computer and gain the inherent advantages of on-line control, data acquisition, and rapid analysis of results.

Reinbold and Pearson [1] described the modifications made to a Perkin-Elmer 141 polarimeter which enabled the acquisition of continuous optical rotatory dispersion data over the spectral region of 650-240 nanometers. The modifications basically consisted of replacing the existing light source of the Model 141 polarimeter with a double grating monochromator and a high intensity xenon light source. More recently, Simon and Pearson [2] reported further modifications done to the Model 141 polarimeter system to convert it into a low cost recording spectropolarimeter. Their modifications include fitting the monochromator with a synchronous drive mechanism and coupling the Model 141 analyzer to a transmitting potentiometer for the continuous recording of optical rotatory dispersion spectra on a multi-speed, multi-millivolt strip chart recorder.

In recent years, the Perkin-Elmer Model 141 polarimeter has been replaced on the market with a modernized Model 241 polarimeter. The Model 241 polarimeter incorporates solid state and digital circuitry for obtaining optical rotatory data which, unlike the vacuum tube circuitry of the Model 141, was ideally suited for interfacing to an on-line microcomputer system. This paper describes the modifications made to a Perkin-Elmer Model 241 polarimeter to both interface it to an on-line microcomputer data system, and convert it to a microcomputer automated scanning optical rotatory dispersion spectropolarimeter with a range of 650-240 nm.

*Author to whom correspondence should be addressed

### Experimental

Figure 1. shows a block diagram of the computer automated scanning spectropolarimeter. Each of the major components shown in Figure 1 will be discussed in detail. Figure 2 shows a photograph of the actual Model 241 polarimeter with some of the modifications visible.

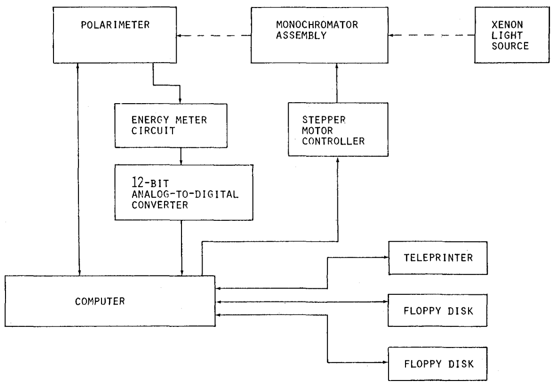

*Figure 1. Block diagram of the computer automated scanning spectropolarimeter.*

*Figure 2. Photograph of the modified Perkin-Elmer Model 241 polarimeter with light shields removed.*

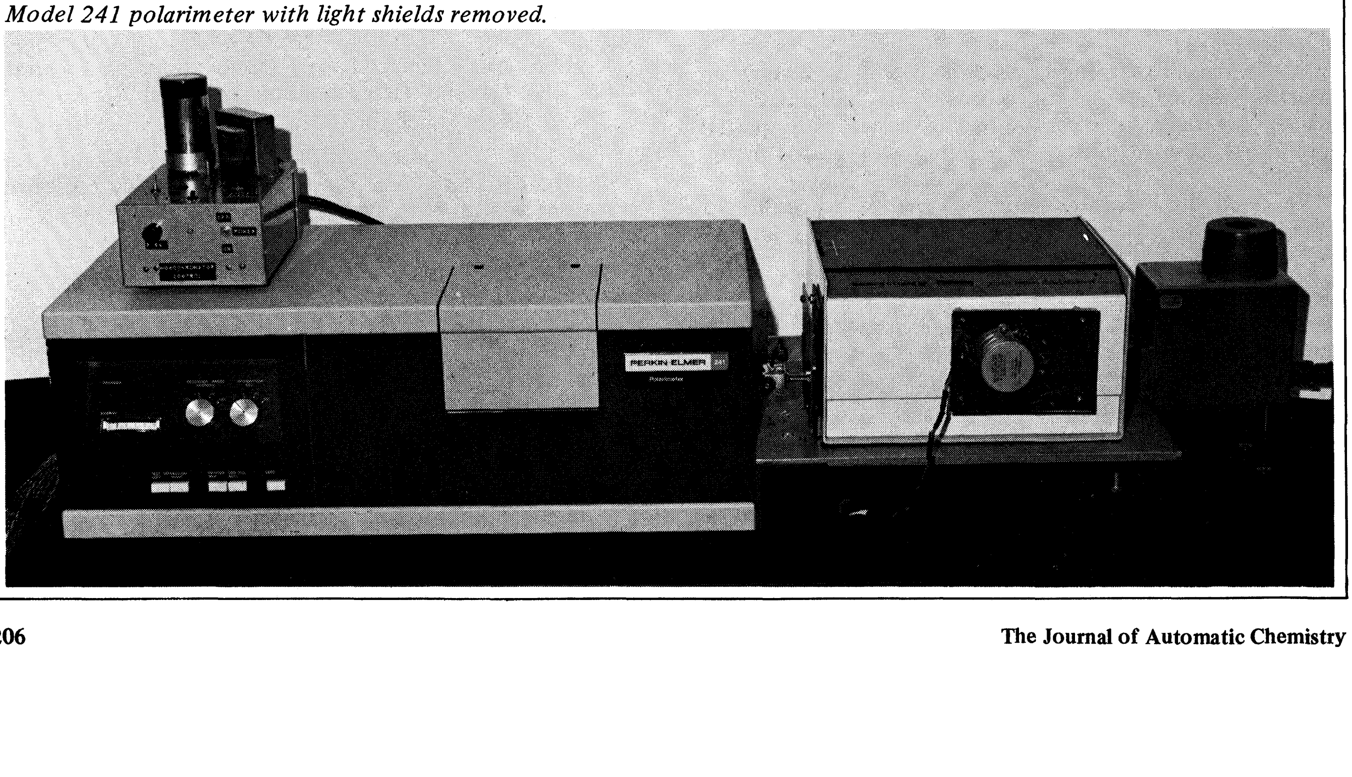

**Computer system**
The computer system used was a MITS ALTAIR 8800A microcomputer system which incorporates the Intel 8080A microprocessor unit. The system has 48K bytes of random access memory (RAM) and 8K bytes of programmable read only memory (PROM). Peripheral input-output devices include dual MITS Model 88-DCD floppy disk drives, a Texas Instruments Silent 700 teleprinter, and a Houston Instruments HI-PLOT digital plotter.

Acquisition and output of digital information to and from the Model 241 polarimeter was accomplished through the use of two Motorola MC 6820 peripheral interface adapters (PIA). The two PIA units, each of which incorporates two independent, selectable, 8-bit parallel input/output ports, are set up such that three of the four ports function for input of parallel data to the computer from the polarimeter. The fourth port functions to transfer control signals from the computer to the polarimeter system.

Analog voltage information from the polarimeter was obtained by connection of the appropriate signal to a 12-bit two's complement, 100 μsec conversion time, analog-to-digital converter (ADC), Datel Inc., Model ADC-D12B. The parallel digital information from the ADC was obtained by the computer with the use of an additional PIA unit.

**Monochromator**
The original light source of the Model 241 polarimeter was removed and replaced with a Bausch & Lomb 250-mm double grating monochromator (Catalog No. 33-86-66) and a Bausch & Lomb 150 watt high intensity xenon light source (Catalog No. 33-86-20-01). An optical bench was fabricated to support and allow for optical alignment of the light source and monochromator with the polarimeter. The actual physical mounting procedure for mating the monochromator and light source assembly to the Model 241 polarimeter was in most aspects identical to that described for the Model 141 [1]. The only major difference was that the overall dimensions of the mounting plate and support bracket are larger because of the larger physical size of the Model 241 polarimeter. The monochromator and xenon light source were obtained from the outdated Model 141 polarimeter.

In order to control the diameter of the beam entering the sample chamber of the polarimeter, a manual two position aperture control was mounted on the monochromator. The aperture control unit, which is identical to the type found in the Model 141 polarimeter, was mounted on a 12.7 x 12.7 cm piece of aluminum plate stock and secured to the exit slit assembly of the monochromator using existing mounting holes and 8-32 bolts. With this modification, the beam size may manually be set to either 6 mm for standard polarimeter cells or 2 mm for micropolarimeter cells.

Control of wavelength position by the computer was accomplished with the construction and attachment of a digitally controlled stepper motor drive assembly to the wavelength drive of the monochromator. The stepper motor chosen for use in the monochromator wavelength drive assembly was a North American Phillips Controls Corporation bidirectional stepper motor, Model K8270-P1 [3].

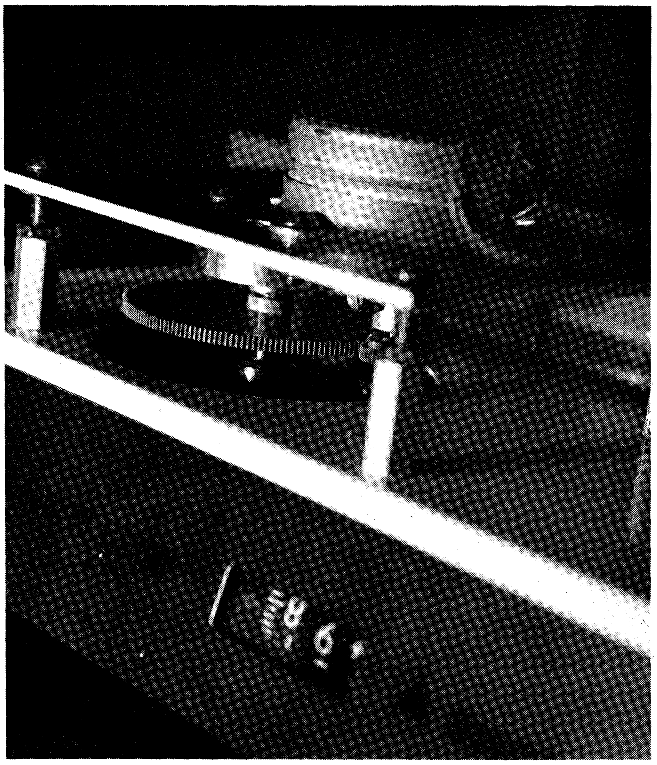

*Figure 4. Photograph of the stepper motor monochromator drive assembly.*

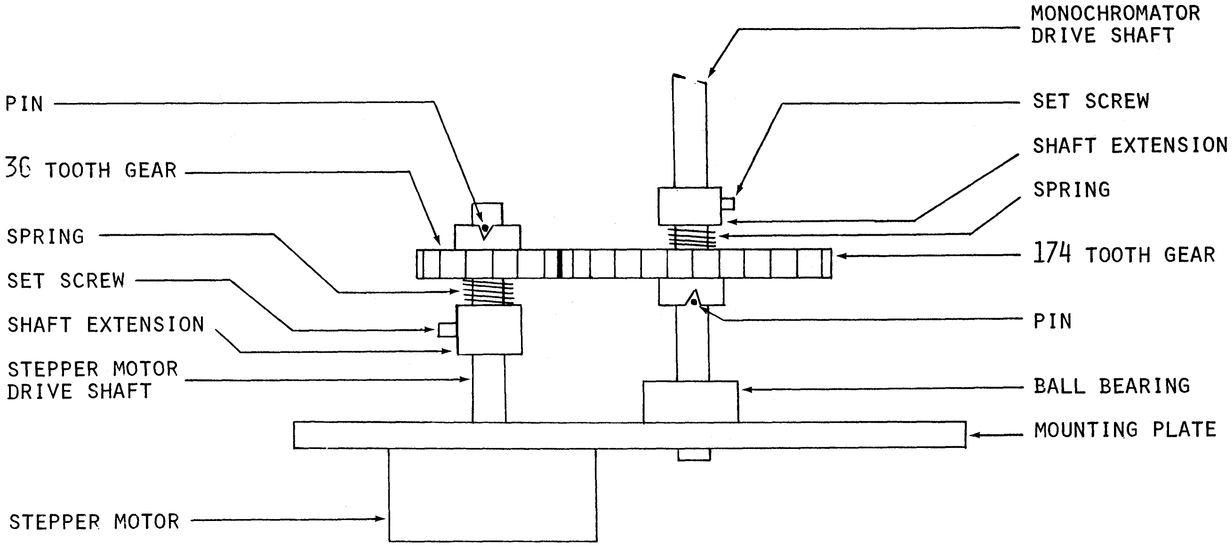

*Figure 3. Diagram of the monochromator reduction gear drive assembly.*

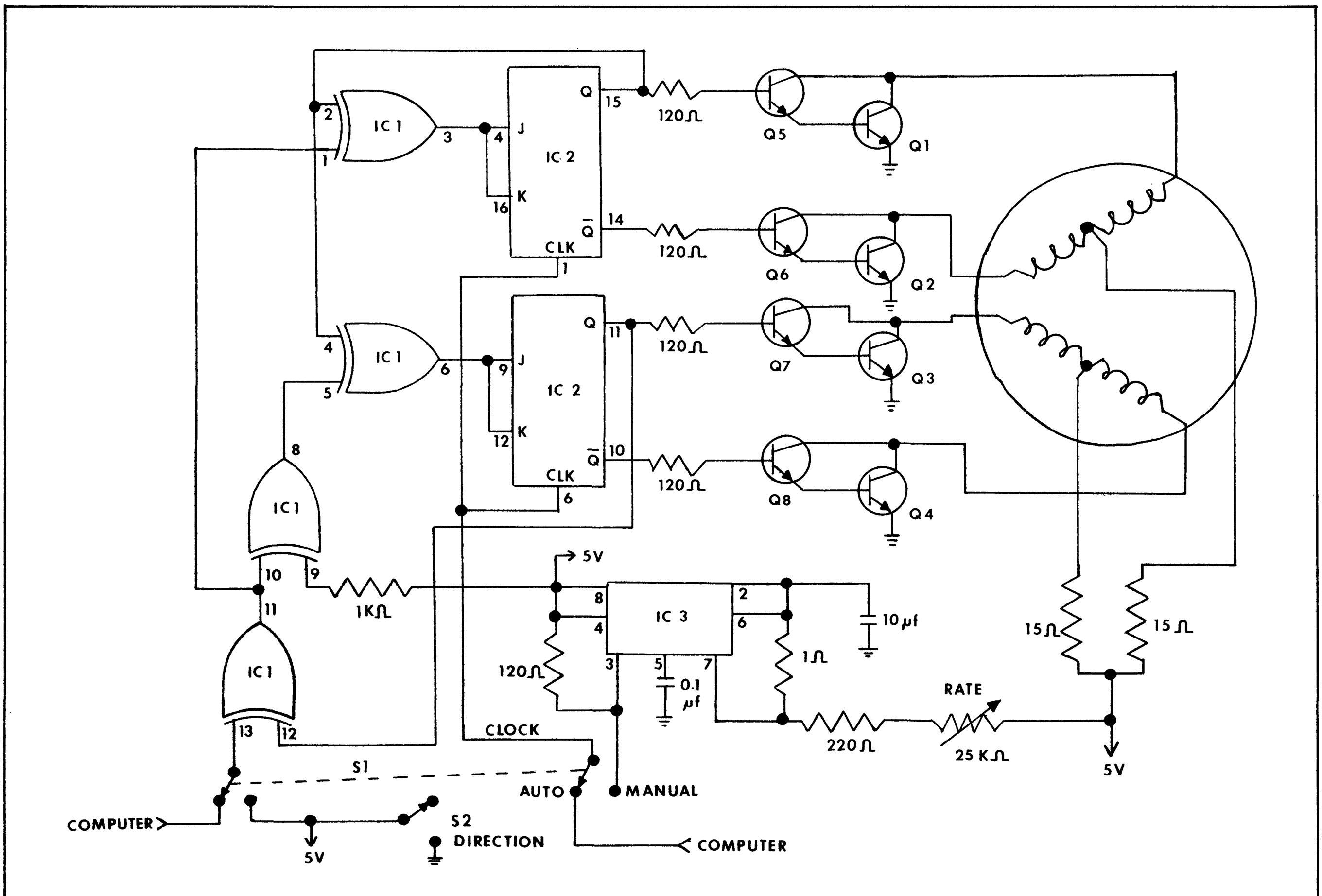

*Figure 5. Schematic diagram of the stepper motor translator control circuit.*
*IC1 = SN7486*
*IC2 = SN7476*
*IC3 = 555*
*Q1-Q4 = 2N3055*
*Q5-Q8 = 2N3394*

This unit was chosen because of its compact size, 7.94 x 6.45 x 5.72 cm overall maximum dimensions, single +5 volt power requirements, and low cost. The stepper motor is bidirectional having a step angle of 7°30′ with a total of 48 steps per revolution, a maximum speed of 300 steps per second, and a holding torque of 936 g-cm. With only a total of 48 steps per revolution, direct coupling of the stepper motor to the wavelength drive shaft would not yield satisfactory resolution. One revolution of the monochromator drive shaft covers approximately 33 nanometers, giving only approximately 0.7 nanometers per step if directly coupled. Figure 3 shows the reduction gear drive assembly that was constructed and fitted to the monochromator wavelength drive shaft. The original manual wavelength drive knob of the monochromator was removed and a 6.35 cm extension with ball bearing was connected to the existing drive shaft using set screws and a coupling. This shaft was turned with a 174 tooth gear attached to the shaft through a spring loaded pin-slip clutch. The ball bearing on the monochromator shaft was necessary to prevent wobble and excessive friction in the monochromator. The stepper motor drives a 36 tooth gear that meshes with the 174 tooth gear. The motor drive shaft was also linked to the 36 tooth gear with a spring loaded pin-slip clutch. Both gears are Cary Model 15 spectrophotometer drive gears. A 10.2 x 15.2 cm aluminum plate was fabricated to hold the stepper motor and bearing assemblies. This mounting plate was secured to the housing of the monochromator with four adjustable 3.8 cm standoffs. Alignment of the mechanical parts was accomplished by elongated and oversize holes in the mounting plate. Figure 4 is a close-up photograph of the actual stepper motor drive assembly secured to the monochromator. The reduction gear drive assembly yields a 0.14 nanometer per step resolution with the existing gears.

The stepper motor direction and speed was controlled either by the computer or manually by use of a special control circuit. Figure 5 shows a schematic diagram for the stepper motor translator control circuit. This circuit functions to energize or de-energize the four coils of the stepper motor in the correct sequence for clockwise or counter-clockwose rotation. Darlington transistors drive one each of the paired motor coils depending on the state of the corresponding flip-flop. The flip-flops are alternately switched in response to each clock pulse. The direction input determines the flip-flop sequence. In the manual mode of operation, the direction was selected by the setting of switch S2 and the clock pulses are generated locally and controlled by the 25k ohm variable resistor. In the computer controlled mode, switch S1 connects both the direction and clock signal inputs to two lines of the computer PIA. With appropriate software, the computer can control both direction and speed of a scan, the latter through the generation of clock pulses at an appropriate rate.

### Polarimeter

The Model 241 polarimeter measures optical rotation by the automatic null point principle. Monochromatic light passes through a linear polarizer, the sample chamber and a servo-controlled analyzer to the photomultiplier. The optical null point is achieved when the polarizer and analyzer polarization planes are oriented orthogonal to one another. When an optically active sample is introduced into the beam path, the analyzer is rotated in the appropriate direction by the servo system until optical null is again achieved. The angle of rotation required to achieve optical null is measured by the Model 241 polarimeter by reading and summing for a selectable period of time the output of an optical pulse generating circuit attached to the servo controlled analyzer. The measured angular rotation is given by an illuminated digital display on the front of the instrument in the fixed form ±XX.XXX degrees. Corresponding TTL-BCD signals are available at a 30-pin connector for a printer option, and were used for data acquisition by the microcomputer [4]. Data was routed to the microcomputer parallel input ports via a 24 conductor flat cable. Twenty of the wires carry the BCD code for each of the five digits of the rotation value. One wire carries the sign of the result. Another carries a 20 msec status pulse from the polarimeter indicating stable data is present. This pulse occurs simultaneously with the display update. The Model 241 polarimeter originally had factory selected integration times of 1, 5, 20 and 50 seconds, allowing a maximum of only one computer acquired data point per second. This was not considered a fast enough response, especially when using the system in kinetic research. At the suggestion of the manufacturer's engineers, the frequency of the oscillator within the polarimeter which drives the digital display and integrating circuitry was increased by a factor of ten, therefore decreasing integration times by a similar factor.

Computer data acquisition was performed using the 0.1 second integration mode. The computer with appropriate software can simulate any of the original integration times or set up new ones as desired.

The Model 241 incorporates a circuit which automatically balances the electronics for stable and precise operation at low light levels. The balancing procedure stops the analyzer servo system for five seconds every three minutes. During this time the digital display and BCD output maintain the current reading, however, the data ready signal will continue to occur at the selected integration rate. In manual operation, the operator is alerted to this hold condition by a continuously lit update lamp on the front of the polarimeter. When this condition occurs, rotational values must be ignored. At the end of the balance period, the analyzer will continue to approach optical null. For the computer, valid data was available only when the update lamp was off and the data ready signal was high. The lamp circuit was tapped at point A in Figure 6 to obtain a computer status signal that is compatable with TTL levels. Only when the data ready signal and the signal from point A are true is the data valid. This is the 23rd wire of the flat cable. The 24th is the common.

The Model 241 polarimeter incorporates an energy meter which indicates the light intensity at the photomultiplier. This 0-100 μA meter, driven by the output of a demodulator circuit from the photomultiplier, was connected to the computer ADC through an Analog Devices AD520K differential input instrumentation amplifier circuit [5]. This circuit (Figure 7) was constructed on a 2.54 x 5.08 cm piece of circuit board and mounted inside the back plate of the polarimeter. The properly scaled 0-5 V output of the amplifier was connected to the ADC with shielded coaxial cable. The ± 15 volts required by the circuit was obtained from the polarimeter. Using previously obtained calibration data, the computer can monitor the energy level of the light to determine when the energy level is too low to yield valid results.

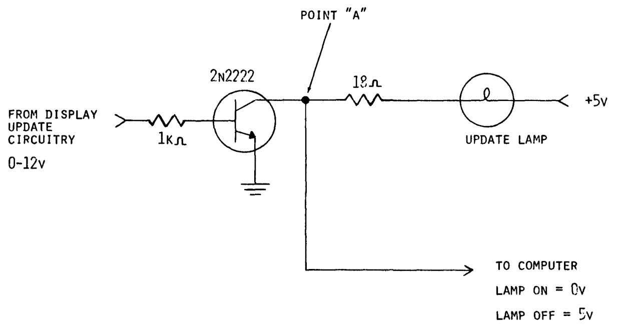

*Figure 6. Schematic diagram of the signal used along with the data ready signal to determine when data is valid.*

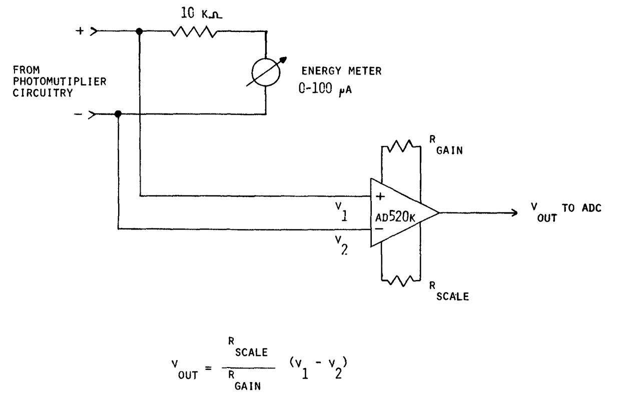

*Figure 7. Schematic diagram of the amplifier circuit for interfacing the polarimeter energy meter to the ADC.*

**Software**

The programs to obtain data from the spectropolarimeter and to control the stepper motor were written in assembly language and exist as subroutines in a little over 300 bytes of PROM. Application programs were written in MITS DISK Extended BASIC (Ver 4.0) with linkage to the assembly routines through user defined subroutine functions [6].

The software was divided into two parts, the section that performs the scan, and the section that analyzes the data. Figure 8 shows a generalized flow diagram for the scan software.

Upon start of the program, the user is asked by a message output on the teleprinter if a scan is desired. If the user responds no, the program proceeds to the data analysis section. If yes, the first section initializes the parallel input/output ports through a call to a short assembly language subroutine which programs the MC6820 PIA chips. No data is passed to or received from this routine. Next, the user is prompted to enter the required scan information through the teleprinter. The user enters such data as an abstract, initial wavelength, final wavelength, number of points to average per reading, wavelength increment between points, molecular weight of the sample, concentration and cell path length. After all the data is entered, the user is asked to verify the correctness of the data. If incorrect, the user has the chance to repeat the entry process. Next, the user is requested to manually set the monochromator to a setting of approximately 15 nanometers greater than the desired starting point and then to input this exact value. The manual step is required so that the software knows the current monochromator position to allow for gear and monochromator backlash. The software computes the number of steps required to reach the desired starting point. The actual movement of the stepper motor is accomplished by repetitive calls to an assembly language subroutine which performs one step in the selected direction with each execution. A signed 16-bit integer value passed to the subroutine determines the direction and speed of the step. A positive value will cause the wavelengths to increase and a negative value will cause a decrease. The absolute value of the number is used as a counter in a software time delay loop started after each step is initiated. The larger the number, the longer the delay between each successive step. Using this routine, and a loop in the main program, rates of between 3 and 300 steps per second can be achieved.

Once the initial wavelength is set by the program, the user is asked to verify that the initial wavelength has been correctly achieved. If for some reason the monochromator has been set wrongly, the user may re-execute the routine. Next, the user is required to enter a unique spectrum name. All data obtained during the scan, as well as the previously entered data, are stored on the disk as a sequential file under this name. Figure 9 shows the file format used to store the scan data. Once the file has been created on disk, the scanning procedure can begin. The value of the rotation data is obtained by calling an assembly language subroutine which reads the BCD values for the data point and converts them into a signed 16 bit integer value which is passed back to the main program. By this method, rotation values of up to ± 32.767 degrees may be read from the polarimeter. This routine checks both the data ready signal and the hold status lines for valid data. Should a hold condition occur during acquisition of data, the routine waits until the hold condition has terminated and then delays 4 seconds for the analyzer to achieve a stable reading before returning to the main program with a value. By using the assembly language routine, it is possible to obtain the data points as fast as the polarimeter can present them at 100 millisecond intervals. With the use of a software loop which calls this acquisition routine a selected number of times, the desired number of points can be collected and averaged at any given wavelength. Upon return from the assembly language routine, the energy meter is checked to verify that sufficient energy is present at the photomultiplier to yield valid data. This limit is experimentally determined and fixed, but may be changed as required by altering the value of a variable within the program. If the energy level is acceptable, the data point is stored along with a single character flag indicating a valid data point. If the energy is below the acceptable limit, the data point is stored, but with a different single character flag indicating low energy.

Next, a check is made to see if all the data points have been acquired. If not the number of steps required to reach the next wavelength increment is computed and the stepper motor is driven this number of steps. This sequence is repeated until the scan is complete, at which time the file is closed and the user may either perform more scans or go into the data handling sections of the software.

The data analysis features of the software include both computational and display capabilities. Any one of a number of routines may be accessed by the user. The available routines include the following:

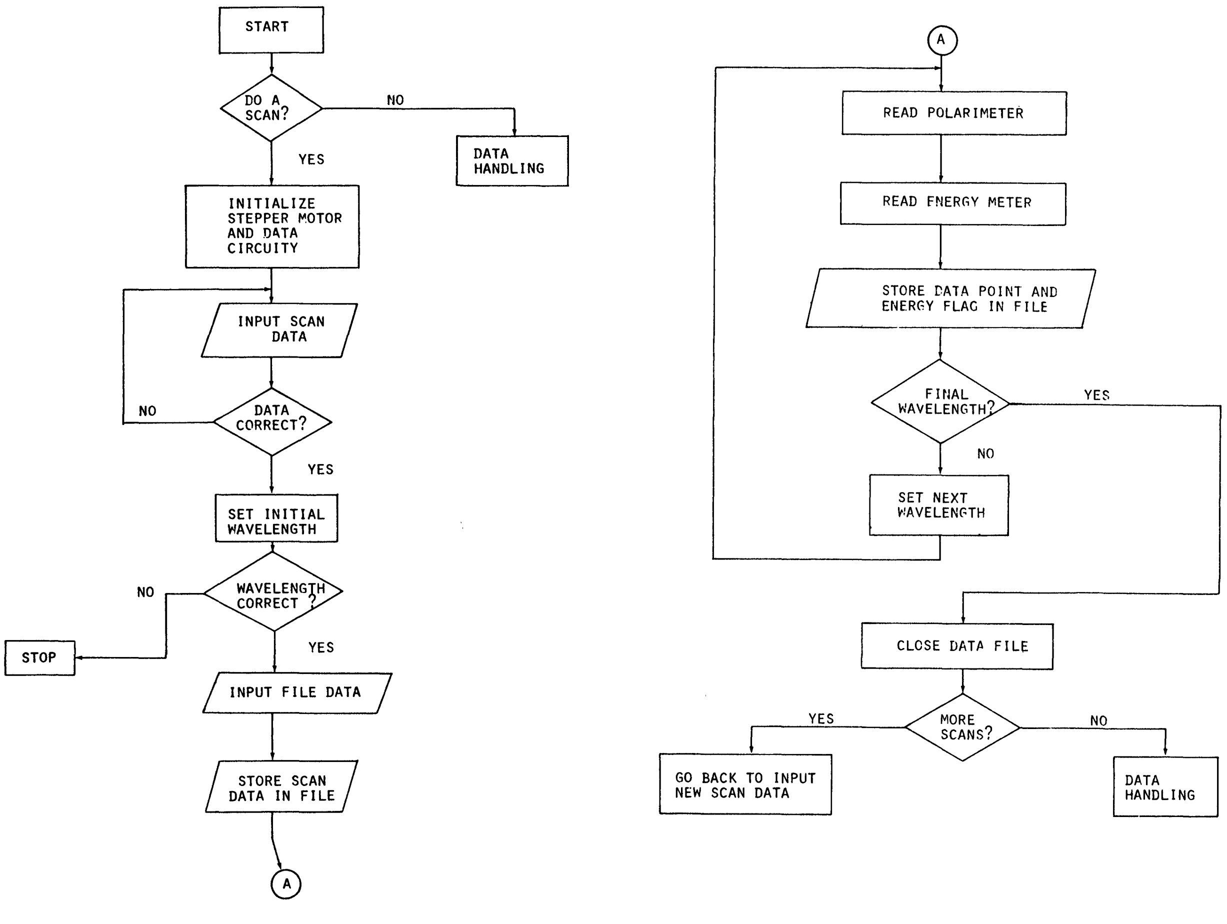

*Figure 8. Generalized flow diagram of the scan software.*

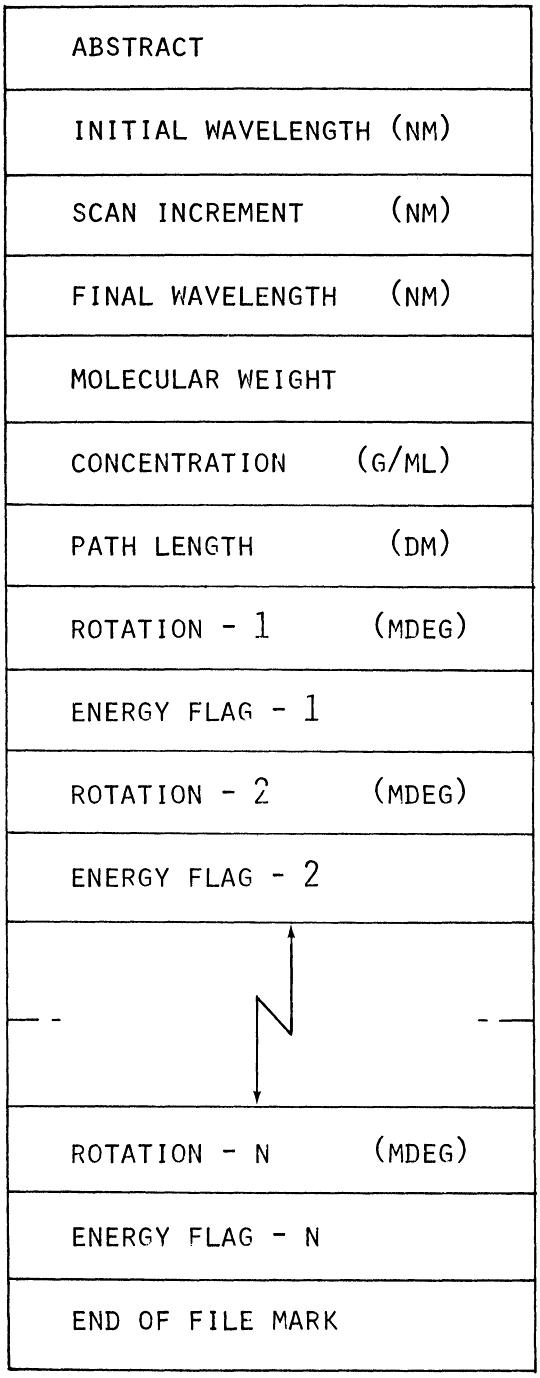

*Figure 9. Disk file format of the stored scan data.*

(1) *Teleprinter plot of stored spectral data.* In this routine the user selects the file name of the spectrum to be plotted and the plot increment. The file is read from the disk and printed on the teleprinter in a scaled point type of plot using a 60 character wide 'floating' origin field. Wavelengths and observed rotations are presented to aid in interpretation. Figure 10 shows a typical representation of an ORD spectrum of $(-)_{546}$- $[Co(R(-)pn)_3]\ I_3$ plotted at 10 nanometer intervals.

(2) *Difference spectrum calculation.* This routine computes the difference between any two spectra stored on disk on a point-by-point basis. The spectra must match each other in scan increment and wavelength regions, otherwise no computation will be performed. The user enters the two file names for the spectra which are to be subtracted. The resulting difference spectrum is stored as a separate user named file on the disk. This routine is used extensively for computing baseline corrected spectra.

**Table I. Comparison of observed and calculated ORD values for sucrose in water at 20 °C, c = 3.01 grams/litre, path length = 1 decimeter.**

| Wavelength, nm | Calculated, α | Observed, α | Error, deg | Error, % |
|---|---|---|---|---|
| 650 | +0.162 | +0.164 | +0.002 | +1.23 |
| 640 | 0.168 | 0.170 | +0.002 | +1.19 |
| 630 | 0.173 | 0.174 | +0.001 | +0.58 |
| 620 | 0.179 | 0.178 | −0.001 | −0.56 |
| 610 | 0.186 | 0.184 | −0.002 | −1.08 |
| 600 | 0.192 | 0.193 | +0.001 | +0.52 |
| 590 | 0.199 | 0.201 | +0.002 | +1.01 |
| 580 | 0.207 | 0.207 | 0 | 0 |
| 570 | 0.215 | 0.213 | −0.002 | −0.93 |
| 560 | 0.223 | 0.222 | −0.001 | −0.45 |
| 550 | 0.232 | 0.232 | 0 | 0 |
| 540 | 0.241 | 0.241 | 0 | 0 |
| 530 | 0.251 | 0.250 | −0.001 | −0.40 |
| 520 | 0.262 | 0.261 | −0.001 | −0.38 |
| 510 | 0.273 | 0.273 | 0 | 0 |
| 500 | 0.285 | 0.283 | −0.002 | −0.70 |
| 490 | 0.298 | 0.296 | −0.002 | −0.67 |
| 480 | 0.312 | 0.309 | −0.003 | −0.96 |
| 470 | 0.326 | 0.326 | 0 | 0 |
| 460 | 0.342 | 0.342 | 0 | 0 |
| 450 | 0.360 | 0.358 | −0.002 | −0.56 |
| 440 | 0.378 | 0.378 | 0 | 0 |
| 430 | 0.398 | 0.397 | −0.001 | −0.25 |
| 420 | 0.420 | 0.419 | −0.001 | −0.24 |
| 410 | 0.444 | 0.442 | −0.002 | −0.45 |
| 400 | 0.470 | 0.470 | 0 | 0 |
| 390 | 0.498 | 0.498 | 0 | 0 |
| 380 | 0.529 | 0.528 | −0.001 | −0.19 |
| 370 | 0.564 | 0.564 | 0 | 0 |
| 360 | 0.602 | 0.602 | 0 | 0 |
| 350 | 0.644 | 0.643 | −0.001 | −0.16 |
| 340 | 0.691 | 0.690 | −0.001 | −0.14 |
| 330 | 0.744 | 0.745 | +0.001 | +0.13 |
| 320 | 0.803 | 0.803 | 0 | 0 |
| 310 | 0.871 | 0.872 | +0.001 | +0.11 |
| 300 | 0.948 | 0.951 | +0.003 | +0.32 |
| 290 | 1.038 | 1.039 | +0.001 | +0.10 |
| 280 | 1.141 | 1.143 | +0.002 | +0.18 |
| 270 | 1.263 | 1.264 | +0.001 | +0.08 |
| 260 | 1.407 | 1.406 | −0.001 | −0.07 |
| 250 | 1.582 | 1.579 | −0.003 | −0.19 |
| 240 | 1.795 | 1.799 | +0.004 | +0.22 |

(3) *Derivative spectrum calculation.* This routine computes the average slope between successive sets of three points in a spectrum, the computed slope value being associated with the middle value. The computed spectrum is stored under a separate file name on the disk using the same format as Figure 9, except that the space for rotational values are filled with average slope values.

(4) The maximum and minimum rotational value in any stored spectrum within the defined wavelength region can be retrieved.

(5) For any stored spectrum, the zero axis crossing may be determined in a defined wavelength region.

(6) The specific and molecular rotations for any stored spectrum may be computed for a specific wavelength or for a defined wavelength region.

(7) The entire contents of a spectrum file may be listed in tablular format on the teleprinter.

(8) A routine that determines the percentage error based on an actual baseline corrected sucrose spectrum and values calculated from a one term Drude equation [7] is used for polarimeter standardization.

In any of the above data handling operations those rotational values which were obtained under insufficient light intensity are indicated on output by printing an 'X' immediately after the observed rotation value. Thus, the user always knows when data points are valid or invalid due to energy.

(9) The final data handling routine available is one which will output a stored spectrum file to the HI-PLOT digital plotter. The software allows high resolution plots of spectra as well as alphanumeric labelling and marking of the axes. Figure 11, to be discussed later, is a typical example of the output from this routine.

Other specialized software for performing such analytical techniques as spectropolarimeteric titrations and ligand-ligand exchange kinetics are available for use with this system.

### Solutions

*Standard Sucrose Solution.* This solution was prepared by dissolving 3.01 grams of J.T. Baker ULTREX analyzed grade sucrose in deionized water and diluting to one litre.

*0.01 M Neodymium-R(-)PDTA Solution.* This solution was prepared by combining 1:1 stoichiometric amounts of the standardized metal perchlorate solution with standard disodium dihydrogen R-(-) -1, 2-propylenediaminetetraacetate, buffered at pH 5.0 with acetic acid-sodium acetate buffer and diluted to volume [8].

1.0% Tris (R-(-)-1, 2-Propylenediamine) cobalt (III) Iodide, $(-)_{546}$- $[Co(R(-)pn)_3]I_3$, was prepared by dissolving 0.500 gram of the previously prepared optically active complex and diluting to 50.0 millilitres [9];

### Results

Table 1 shows the evaluation of the results obtained from the standard sucrose solution from 650-240 nm as obtained from this computer controlled system. The error from 650-300 nm is within the ± 0.002° specification of the instrument. The error from 300-240 nm is within the ± 0.2% specification of the instrument for optical rotations of approximately 1.0° or greater. Calculated values for the optical rotations of the standard sucrose solution were obtained from the equation $[\alpha] = 21.648/(\lambda^2 - 0.0213)$ which assumes single-termed Drude equation behaviour [7], using the polarimeter calibration routine previously described (λ is microns, and path length is in dm).

Figure 11 shows the optical rotatory dispersion spectrum of the neodymium-R(-)PDTA complex obtained by the computer automated system. The data for this spectrum was obtained at 0.14 nm increments and has been plotted as a continuous curve. Representative data points obtained from a Cary Model 60 recording spectropolarimeter are also shown in this figure. The extremely sharp f-f transitions of the

| LAMDA (NM) | ROTATION (MDEG) |
|---|---|
| 600 | -7 |
| 590 | -19 |
| 580 | -42 |
| 570 | -74 |
| 560 | -119 |
| 550 | -182 |
| 540 | -266 |
| 530 | -344 |
| 520 | -348 |
| 510 | -195 |
| 500 | 183 |
| 490 | 676 |
| 480 | 1142 |
| 470 | 1403 |
| 460 | 1349 |
| 450 | 1100 |
| 440 | 789 |
| 430 | 568 |
| 420 | 471 |
| 410 | 458 |
| 400 | 493 |
| 390 | 537 |
| 380 | 577 |
| 370 | 633 |
| 360 | 688 |
| 350 | 706 |
| 340 | 717 |
| 330 | 800 |
| 320 | 1096 |
| 310 | 1197X |
| 300 | 1205X |
| 290 | 1208X |
| 280 | 1211X |
| 270 | 1218X |
| 260 | 1225X |
| 250 | 1251X |

*Figure 10. Teleprinter plot output of the optical rotatory dispersion spectra for the $(-)_{546}$- $[Co(R(-)pn)_3]I_3$ complex.*

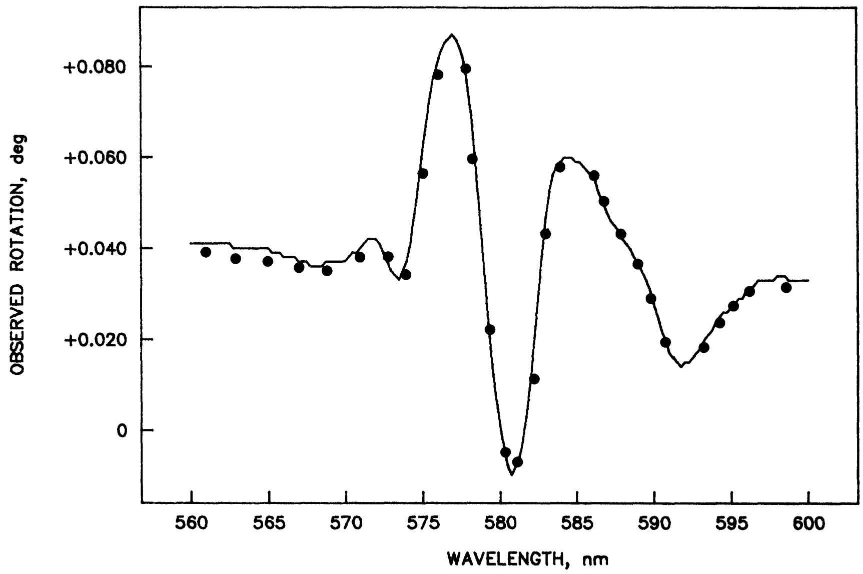

*Figure 11. Optical rotatory dispersion spectra of the neodymium-R (-) PDTA complex*
*– Computer acquired spectra*
*• Selected data points from Cary Model 60 recording spectropolarimeter*

neodymium-R(-) PDTA complex are very clearly resolved by the data obtained from the computer automated system, showing four peaks and troughs which occur over the 25 nm region of this spectrum. The overall accuracy of the data obtained is excellent, as can be seen by comparison with the data obtained from the Cary Model 60 recording spectropolarimeter.

### Discussion

The modifications described in this paper have resulted in a unique computer automated scanning optical rotatory dispersion spectropolarimeter which has a range of 650-240 nm and a number of attractive features. As previously reported (1,2), the Bausch & Lomb monochromator, containing a double-grating modified Czerny-Turner mounting with its low stray light characteristics and excellent dispersion, allow the entire spectrum to be obtained without the need of additional operations on the optical system and subsequent changes in sensitivity. The high intensity xenon lamp source can be used over the entire wavelength range of 650-240 nm, therefore eliminating the need to change lamps to cover the spectral region.

The modifications installed to allow for control of the scanning and data acquisition by the microcomputer result in the rapid and easy acquisition and analysis of optical rotatory dispersion data. The advantage of having a spectrum run by the computer, and having the data digitized and stored for future recall on a disk in a computer readable format, is that time and effort is saved. Calculations and data retrieval can be performed immediately at the completion of a scan, or at any time in the future, without a great deal of effort. With a computer to both perform the scan and analyze the data, an extremely powerful, yet easy to use, scanning spectropolarimeter system was obtained, which excluding the cost of the existing microcomputer and monochromator systems, required less than $400 to construct.

REFERENCES

[1] P.E. Reinbold and K.H. Pearson, *Analytical Chemistry,* 1971, 43, 293.

[2] S.J. Simon and K.H. Pearson, *Analytical Chemistry,* 1973, 45, 620.

[3] "Stepper Motor Series 82700", North American Phillips Controls Corporation, Cheshire, Conn. 06410, U.S.A.

[4] "Model 241 & 241MC Polarimeters", Perkin-Elmer Corporation, Norwalk, Conn. 06856, U.S.A.

[5] "AD520 Integrated Circuit Instrumentation Amplifier", Analog Devices, Norwood, Mass. 02062, U.S.A.

[6] "ALTAIR 8800 BASIC Reference Manual", MITS, Albuquerque, New Mexico 87106, U.S.A.

[7] T.M. Lowry, "Optical Rotatory Power", 1964, Dover Publications, New York, page 131.

[8] P.E. Reinbold, PhD. Dissertation, 1970, Texas A & M University.

